# Moving-Target Defense in Depth: Pervasive Self- and Situation-Aware VM Mobilization across Federated Clouds in Presence of Active Attacks

**DOI:** 10.3390/s22239548

**Published:** 2022-12-06

**Authors:** Yousra Magdy, Mohamed Azab, Amal Hamada, Mohamed R. M. Rizk, Nayera Sadek

**Affiliations:** 1 Electrical Engineering Department, Faculty of Engineering, Alexandria University, Alexandria 21519, Egypt; 2 Department of Computer and Information Sciences, Virginia Military Institute, Lexington, VA 24450, USA; 3 Management Information System (MIS) Department, High Institute of Computer and Information Systems (HICIS), Aboukir High Institutions, Alexandria 21625, Egypt

**Keywords:** cloud federation, virtual machines, blockchain, HARM model, moving-target defense, virtualization, cloud migration

## Abstract

Federated clouds are interconnected cooperative cloud infrastructures offering vast hosting capabilities, smooth workload migration and enhanced reliability. However, recent devastating attacks on such clouds have shown that such features come with serious security challenges. The oblivious heterogeneous construction, management, and policies employed in federated clouds open the door for attackers to induce conflicts to facilitate pervasive coordinated attacks. In this paper, we present a novel proactive defense that aims to increase attacker uncertainty and complicate target tracking, a critical step for successful coordinated attacks. The presented systemic approach acts as a VM management platform with an intrinsic multidimensional hierarchical attack representation model (HARM) guiding a dynamic, self and situation-aware VM live-migration for moving-target defense (MtD). The proposed system managed to achieve the proposed goals in a resource-, energy-, and cost-efficient manner.

## 1. Introduction

**Figure 1:**
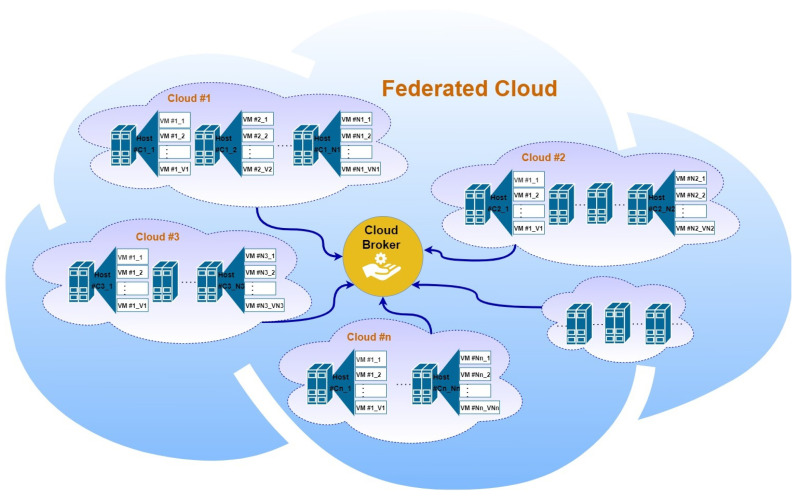
Architecture of federated cloud.

The growth in demand for cloud services presents considerable challenges for cloud providers to meet the requirements of end users and satisfy their expectations [1]. Accordingly, cloud computing patterns have shifted from independent, isolated, heterogeneous providers to collaborative federated clouds. Federated clouds provide a scalable and cost-efficient service to their users, with elastic inter-cloud workload balancing and extended resource sharing, as shown in Figure 1. However, sharing heterogeneous cloud resources among random users can expose cloud VMs to different types of attacks. Resourceful attackers can exploit the limited situational awareness of the federated cloud environment and find a weak entry point in one of the participating clouds to use as a launch pad for pervasive attacks. With a federated elastic migration strategy, an attacker can drive its target (EX. VMs) to such a compromised environment and control its operation [2].

A good example is cross side-channel attacks that exploit isolation vulnerabilities to cross the logical boundaries separating VMs sharing the same physical host resources. Attackers usually exploit their presence on the same host hosting the victim’s virtual machine, construct a side-channel attack and access sensitive data [3,4].

Literature reports indicate that even modern clouds are exposed to different vulnerabilities that could attract attackers [5], with static targets being more vulnerable to attack and eavesdropping than mobile ones. Traditional security approaches cannot keep up as attackers become more intelligent and powerful. Therefore, proactive security mechanisms have been introduced to increase attacker uncertainty, complicate target tracking and attack strategy deployment [6]. Moving-target defense (MtD) has emerged as an effective proactive security mechanism that makes a system unpredictable for attackers. Moving-target defense (MtD) induces dynamic real-time changes to the cloud’s operational characteristics to evade intrusions and attack attempts.

Researchers have categorized MtD techniques into three types according to the change induced during the defense-provisioning process [7]. These categories are shuffle, redundancy and diversity. Shuffle refers to inducing real-time changes to the virtualized resource configuration, such as, location, network address and routing topology to confuse active attackers. Redundancy refers to creating multiple replicas of the targeted system that can be used in the event of failure or attacks. Replicas actively participate in the system operation or synchronize passive machines [8]. Diversity is used to represent the case where a heterogeneous mix of modules is used to construct the target of the defense. Such heterogeneity is exploited by the MtD to change the active system variant and complicate vulnerability tracking [9].

Many researchers have proposed effective security solutions that rely on one of the MtD categories mentioned above. Others have presented enhanced solutions by combining different MtD categories, including shuffle and diversity MtD techniques, to improve the VMs’ security level on the cloud. Based on [10], most of the MtD techniques presented rely on randomized decisions with limited self- or situation-aware guidance of the defense-provisioning process. Furthermore, researchers who have presented MtD algorithms have applied continuous changes in the targeted system regardless of the presence of threats, with no consideration of potential attack points or risk indicators within such systems. The continuous induced change in the cloud consumes too much power and increases service downtime, which may waste users’ time and increase service costs for the user and service provider [11]. Migrating a virtual machine involves suspending VM execution at the source host, transferring the VM state to the destination host and resuming VM execution at the target. The VM migration process can result in extended downtime [12].

Consequently, this paper presents an adaptive MtD modeL(AML), a novel virtual machine mobility framework to enable live cross-cloud migrations of running machines for moving-target defense (MtD). The AML MtD strategy design relies on real-time quantification of risk presented as a multi-objective optimization problem. The presented MtD model represents a risk motive for change which depends on real-time risk calculations of the hosts and VMs deployed in the federated cloud. As VM migration is costly and consumes time, AML triggers VM migration decisions based on their risk level, indicating the presence of threats.

VM risk calculation depends on the exploitability and impact of targeted vulnerabilities on the VM operation. The AML prefers to migrate VMs with high-risk values vs. those with lower-risk values. The AML real-time VM risk calculations will guide the AML to find a suitable host with a low risk to host the migrated VMs. Cloud users’ applications are deployed in a distributed or centralized manner. Highly dependent distributed applications might result in whole-system failure if one of the applications’ VMs stops working. To tackle this problem, the cloud environment allows users to request replication of these critical VMs in different clouds when registering the cloud services. The VM criticality level is an indicator used to reflect the implemented level of module diversity and the number of application replicas. Furthermore, for VMs with low tolerance to failures, such as those hosting sensitive applications, including real-time applications, migrating these VMs could cause a stop in the whole application, with loss of power and time until it resumes working normally.

Consequently, the AML considers the critical nature of VM-hosted applications in migration decision-making. The AML prompts the migration when it detects a VM with high-risk and low criticality factors. The AML builds the migration strategy for VMs, taking into consideration the high-risk and criticality factors of the VM, neighbouring VMs, and the underlying host. The AML will consider migrating the neighbour VMs of a critical target, if they are considered high-risk, to maintain the total risk value of the host under a certain threshold. For time-sensitive applications, the AML considers using containers instead of VMs. Containers offer an alternative virtualization method, making them more lightweight and portable. The migration duration depends on the application characteristics and the memory footprint of the application hosted on the container [13,14]. However, the migration of containers has recently been widely used to minimize downtime of the migrated application, unlike for VM migration. We have assumed that downtime-sensitive applications will use container-based virtualization instead of full VM virtualization. Doing so helps the AML to defend the application with limited or no impact on its operation.

To ensure the privacy of hosted VMs and cloud data, blockchain technology is used to support the AML’s operation. The blockchain enables transparency and immutability and offers an additional layer of security to the migration strategy metadata.

In the AML, security is not the only targeted objective during migration strategy design. Maximizing resource utilization is one of the vital objectives that impact the cloud quality of the service and the operational cost. The AML aims to maximize resource utilization within a federated environment by enabling cross-cloud VM/container consolidation during the migration strategy design.

However, VM consolidation can easily increase the risk level of the cloud hosts. The AML seeks to balance the targeted objectives towards minimizing the risk of attacks and failures, while maximizing cloud resource utilization.

The main contributions of the paper are summarized as follows:

1. Presenting an adaptive moving-target defense model relying on a self- and situation-aware decision-making mechanism.
2. Enabling security-aware, virtual machine consolidation for efficient resource utilization and improved energy harvesting.

The remainder of this paper is organized as follows: Section 2 presents a detailed literature review of federated clouds and previous research that has been undertaken to tackle and solve the security problem. Section 3 details the operation flow of the system. Section 4 introduces the proposed system. Section 5 presents the threat model that we seek to mitigate in this paper. Section 6 presents the system mathematical model used to analyze the federated cloud to enable virtual machine migration. The results are provided in Section 7. Finally, the conclusions and ideas for future work are presented in Section 8.

## 2. Related Work

Cloud computing technology in the field of high-performance distributed computing represents a milestone as it provides shared computing and storage resources as a service upon a user’s request [15]. A service provider’s main requirement is the productive utilization of available resources because it is challenging to provide on-demand resources to users in the optimal manner to improve performance and achieve faster computation time. Moreover, with the advent of computers, scheduling problems that arise in industry through the use of technology have received significant attention in numerous fields.

Conventional cloud computing systems possess limitations. It is difficult for users to switch between various cloud providers due to the lack of a standard architecture. There is no standard metering system for cloud computing services. Metering systems collect metrics about users’ access level to the cloud, their use of computing resources, and specific information about the apps and processes that users run. Some researchers have proposed metering modules for system metric calculations to ensure the end user’s transparent and easy access to cloud computing resources [16,17].

Cloud providers find it difficult to maintain performance transparency due to changing user requirements, varying loads and limited resources, especially for small and medium providers. A federation of clouds is a possible solution to these problems [17].

Federated cloud computing expands the scope of cloud services by pooling together resources from related cloud infrastructures [18].

Although cloud computing has many advantages, security threats can prevent cloud users from making use of them. In [19], many of the security threats and security attacks, with their associated mitigation techniques, were discussed. Some of the attacks addressed included structured query language (SQL) injection attacks, man-in-the-middle attacks, denial-of-service (DoS) attacks and hypervisor attacks. However, the analysis was limited in terms of implementation of the mitigation techniques presented.

The authors of [8] surveyed MtD strategies and considered the randomness, diversity, unpredictability, and uncertainty introduced into a system after applying MtD techniques against some cyber attacks. The cyber attacks studied were code-injection attacks, distributed denial-of-service (DDoS) attacks, computer worm attacks and reconnaissance attacks. The use of different MtD strategies helped in mitigating these attacks. Reconnaissance [8] is considered the initial phase of almost all attacks. During the reconnaissance phase, attackers utilize various techniques and automated tools to gather information about the target system, such as the OS types, running services and protocols, and open ports for potential vulnerability exploitation and attacks. A system with an enabled MtD strategy is effective because the random changes in the system applied by the MtD technique will invalidate the information that the attackers obtained previously. Although changing host IP addresses by applying MtD techniques can be an effective way to prevent the spread of computer worms, several studies have focused on investigating moving-target defense (MtD). According to [20], the research on MtD can be classified into three areas: MtD theory, MtD strategy and MtD evaluation. The authors of [20] suggested that applying MtD depends on choosing the appropriate time to apply the MtD, the MtD technique, and how it will be applied. However, they did not consider the cost or period of the MtD movement. They only explored the common properties of the MtD (e.g., movement selection, movement strategy and movement time) that should be achieved when an MtD technique is applied.

In [21], a blockchain-based routing and addressing mechanism, BMtD, was proposed to ensure anonymous capsule migration in federated-cloud environments. BMtD mitigates co-resident attacks using shuffle MtD. Using blockchain, BMtD hides the virtual machine’s public IP that attackers depend on to trace the target virtual machine. The automated blockchain-based anonymous federated cloud provides secure capsule-hosting and migration. The decision to enact capsule migration is random, depending on the user’s migration request, without continously tracing the machines’ security metrics.

To evaluate the effectiveness of different MTD techniques, including shuffle, diversity, redundancy, or combinations of these, we modeled MTD techniques using a graphical security model named the hierarchical attack representation model (HARM). The HARM model is used to evaluate the overall security of the MtD system. It is used to evaluate how the security metric values change when MTD techniques are applied. MtD techniques can be used independently or combined to obtain more effective results. A diversity technique is used to replace the systems’ components (e.g., a VM, server, programming language, OS, hardware, etc.) with different components. In comparison, the shuffle technique changes the reachability of the VMs in the cloud. At the same time, redundancy enables replication of the system components.

The authors of [22] studied the effectiveness of different MtD techniques using the Harm model. They investigated the effects of combining different MtD techniques, such as diversity, shuffle and redundancy, and evaluated them in terms of security. First, the MtD techniques were deployed and the results compared for security without MtD. The three main MtD techniques, shuffle, diversity and redundancy, were then combined to investigate their effects.

In addition, the results were applied to the E-health cloud model to evaluate the cost of security against the security measurements. Only OS vulnerabilities were investigated to perform the security analysis. However, for the AML system, we investigated both OS vulnerabilities and co-resident attacks by evaluating the security effect of MtD on each to show the effect of applying MtD on different types of attacks. Moreover, we did not combine more than one MtD as this would have increased the cost of the solution and required more time for its application. However, we applied the best MtD technique for each attack. In [23,24], a combination of two MtD techniques was proposed, shuffle and diversity, which minimized the attacks exposed to the cloud. Based on the results obtained, it was concluded that combining both shuffle and diversity can help to improve all security metrics.

Most current research has focused on investigating MtD techniques and combining them to assess the enhancement in security after applying this combination. However, suitable criteria for applying the MtD decision have not been used, rather, evaluation has been based on abitrary judgements.

Continuous change in the cloud consumes much power and increases service downtime, which may waste users’ time and increase service costs for users and service providers. In AML, we trigger VM migration decisions based on their risk security level, reflecting the presence of threats. In addition, the migration decision is based on host utilization.

## 3. Operation Flow

**Figure 2:**
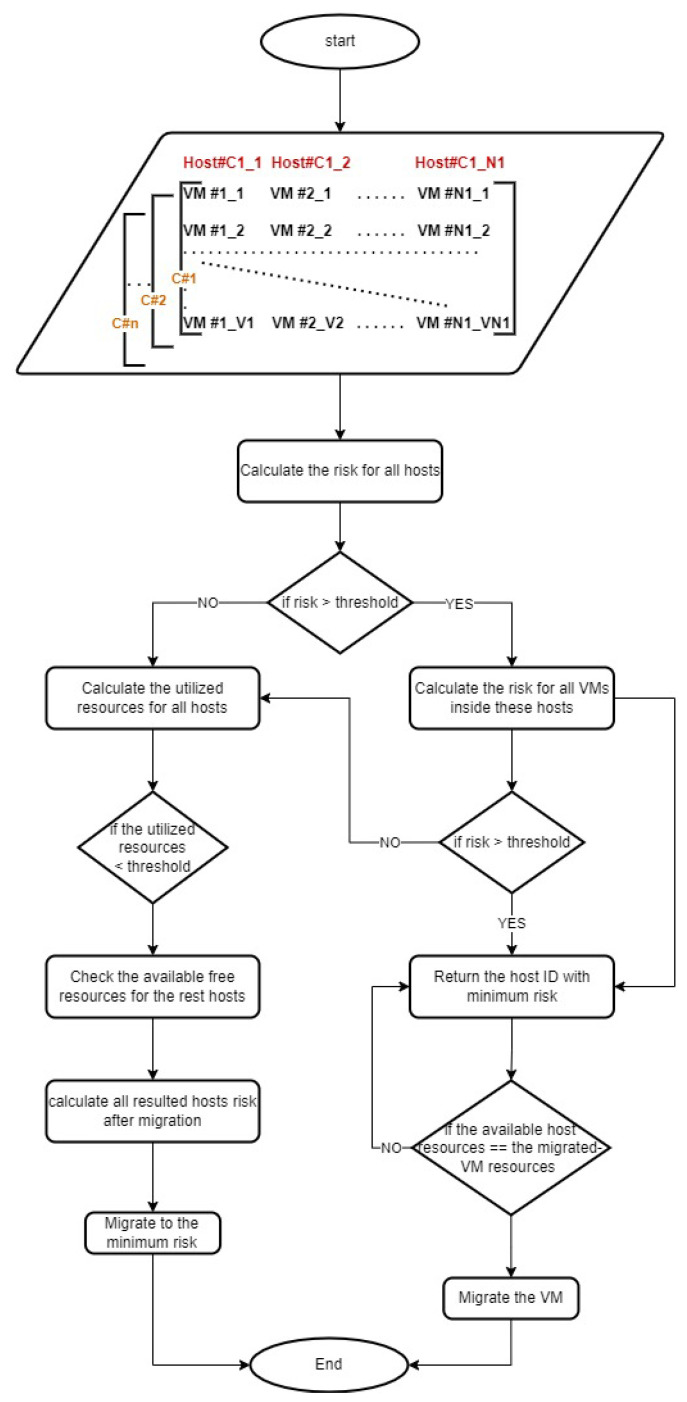
Deployment and migration flow diagram.

For AML, we investigated co-resident side-channel attacks and attacks that target operating system (OS) specific vulnerabilities. The co-resident side-channel attacks only target machines deployed on the same host. The OS attacks affect VMs through a specific attack path that can reach the target machine by exploiting a vulnerability in another VM connected to the target VM. The details of system vulnerabilities leading to the above attacks can be found in the CVE database. The CVE [25] list is a set of records describing specific vulnerabilities or exposure. There are multiple ways to evaluate the severity of a vulnerability. One is the common vulnerability scoring system (CVSS), a set of open standards for assigning a number to vulnerability to indicate its severity. The National Vulnerability Database (NVD) [26] uses CVSS scores to assess the impact of vulnerabilities. CVSS scores range from 0.0 to 10.0, with higher numbers indicating a higher degree of vulnerability, as shown in Table 1. The AML system addresses the impact of these vulnerabilities through a set of modules, such as registration, blockchain-based anonymity, deployment management, risk calculation and cloud modules. The deployment module consists of two sub-modules, a resource utilization checker and a migration sub-module. All the system modules are fully integrated and cooperate to keep the hosted VMs/containers secured. At the same time, maximizing resource utilization enhances the data center energy consumption, minimizing the overall hosting cost. The details of each module are described in Section 4. In this section, we describe the deployment of the newly created VMs/containers and their migration process, as shown in Figure 2.

### 3.1. The Deployment Process

The deployment process starts when a user requests a new hosting for their application with a specific requirement. According to the sensitivity level of the user application shown in Table 2, the deployment management module decides to deploy the user application on a VM or a container.

The risk calculation module calculates the security measurement values, including the risk value, CVSS and other metrics for all hosts and VMs, as described in Section 4. Then, it sends the calculation results to the migration sub-module. At the same time, the resource utilization checker continues to capture all host resource utilization levels in each cloud sub-module and continuously sends them to the migration sub-module.

The migration sub-module analyzes the results of the risk calculation module and resource-utilization checker and determines the target host with a low-risk level and high utilization capacity. Then, the deployment module sends the results to the cloud sub-module to host the new machine.

### 3.2. The Migration Process

As described in the deployment process, the migration sub-module receives the results of the risk calculation module and resource utilization checker and compares them with the predetermined threshold values. The security measurement threshold value is determined based on the CVSS severity rating shown in Table 1. The migration sub-module selects the hosts with medium or higher CVSS severity ratings. Then, it selects the hosted VM with the highest CVSS rating to be migrated.

In the event that there are no hosts with a high risk level, the migration sub-module starts to analyze the host utilization levels and selects the host with a resource utilization level lower than a threshold value. The hosts’ utilization threshold value is determined based on the percentage of used resource capacity. We assume that a resource utilization threshold value of 30% can be easily distributed to the rest of the hosts to maximize cloud resource utilization. The VMs deployed on this selected host are considered to be migrated.

The migration sub-module will consider the criticality level of the selected VM for migration. The criticality level is assumed to be between one and four, as shown in Table 3. The migration sub-module triggers a migration decision for VMs with criticality levels lower than three. It will migrate the high-risk neighbor VMs if the criticality levels for selected VMs are higher than three. The selected VM will be migrated to the host with a low-risk value and a high resource utilization level. Finally, the migration sub-module sends the migrated VM pseudonym ID and the target host to the deployment module. The VM pseudonym ID is a fake name used to hide the real identity of the VM, as described in Section 4. To start moving the VM data, the deployment module starts the migration process by sending to the source and target cloud sub-modules.

## 4. The Proposed System

The proposed system, the adaptive MtD model (AML), proactively mitigates the impact of a known set of attacks that it is challenging to tackle using common defense tools. The AML is a risk-aware model that uses shuffle and diversity MtD techniques to mitigate the impact of different attacks, such as co-residency and attacks that target operating system (OS)-specific vulnerabilities. It applies the MtD by shuffling to the VMs that might be vulnerable to co-residency attacks. Further, the AML applies an MtD strategy based on diversity and shuffle techniques to those exposed to OS attacks.

The presented model aims to keep the hosted machines secure while maximizing the hosts’ resource utilization. Consequently, the AML triggers the VMs’ migration decision, relying on the hosts’ risk calculations and resource utilization levels. Moreover, the presented model considers the critical nature of the VMs during the migration process.

**Figure 3:**
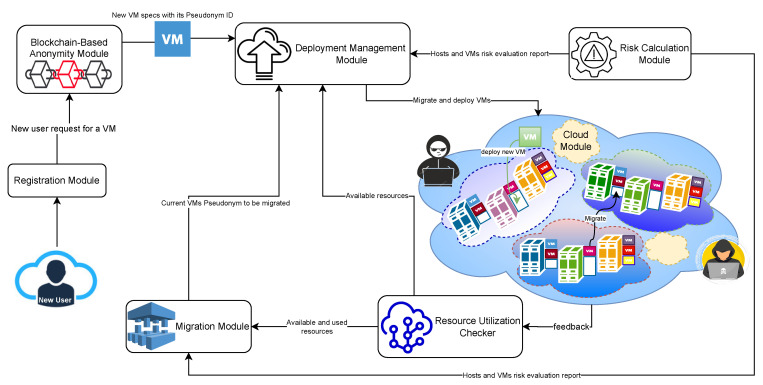
System architecture.

The proposed system consists of five main modules: a registration module, a blockchain-based anonymity module, a deployment management module, a risk calculation module and, finally, cloud modules, as shown in Figure 3. In the following sub-sections, we discuss each of the modules in detail.

### 4.1. Registration Module

The user registers to the cloud asking for a new VM/container with certain specifications and provides sufficient metadata regarding their application’s sensitivity and criticality to begin the VM/container deployment process. The registration module receives the user’s request and assigns a public/private key pair to the new user. Then, it sends the users’ requests to the blockchain-based anonymity module to hide their real identities before starting the VM/container deployment process.

### 4.2. Blockchain-Based Anonymity Module

Blockchain is a fault-tolerant decentralized technology with no single point of failure. Using blockchain with the presented system preserves the privacy of all the hosted VMs’ metadata. After receiving the users’ requests, the blockchain-based anonymity module assigns a pseudonym identification (PID) for each VM deployment request. A pseudonym identification (PID) is a fake name used to conceal the real identity of the VM. Using a PID instead of real identities ensures the privacy of the hosted VMs and of the cloud data.

After that, the AML system generates the users’ transactions containing their public keys, PIDs, and the required specifications that fit their applications. Then, the user transactions are signed with their private key. According to the sensitivity level of the user application, the deployment management module decides to deploy the user application on a VM or a container.

The real identity of the VMs is known to the deployment module only for managing the overall VM deployment and the migration processes.

### 4.3. Deployment Management Module

The deployment management module orchestrates the system interaction process, from receiving the required machine specifications to deploying or migrating the VM/container. Depending on the users’ application, the deployment management module will deploy the application on a VM or a container. Real-time applications are downtime-sensitive applications. Consequently, the deployment module will deploy the application on a container instead of a VM to minimize downtime during migration.

The deployment management module consists of two sub-modules: the resource utilization checker and the migration sub-module. The resource utilization checker captures the hosts’ utilization level in each cloud module to ensure tracking of the host resource usage, which helps in the migration decision. The migration sub-module is responsible for the VMs/containers migration decision. It examines and checks all the risk calculations and criticality values of the VMs along with the host resource usage level. It selects the VM that needs to be migrated and the target cloud host based on its analysis after receiving the results from the risk-calculation module and the resource-utilization checker. The VMs detected with high risk and low criticality factor values will be triggered for migration. However, for VMs found with high risk and high criticality factor values, we can consider migrating the neighbours’ VMs with high risk values to minimize the total risk value of the host. The VM to be migrated will be hosted in a cloud host with a low risk value and high resource-usage levels.

The migration sub-module then sends the pseudonym ID of the current VM to be migrated to the deployment management module to start the actual deployment/migration process. The deployment module will start communications with the target cloud module to begin the VM deployment process. In the case of VM migration, it first communicates with the source cloud module to start transferring the VM data to the new cloud.

### 4.4. Risk-Calculation Module

The risk-calculation module performs all the security measurement calculations, including risk value, CVSS, attack cost, and return-on-attack (RoA) for all VMs and hosts. These security measurement values are calculated based on knowledge of the exploitability indicators and the impact of existing vulnerabilities in the VM environment. The exploitability values represent the characteristics of the vulnerabilities, whereas the impact values indicate the attackers’ ability to exploit VM vulnerabilities to violate security requirements. The exploitability and the impact values of different vulnerabilities can be found in the CVE databases [25]. The risk-calculation module continually updates its calculation results based on any change in the system and sends this feedback to the migration sub-module to support the migration decision.

### 4.5. Cloud Module

The cloud module represents the clouds in the federation. Each cloud in the federation is represented by a cloud sub-module hosting the set of VMs/containers. The cloud module hosts the VMs based on the deployment/migration decisions received from the deployment-management module. Each cloud sub-module is responsible for reserving required resources for launching the VMs/containers and assigning these resources to them. To check the resource availability of every host in the federated cloud, the resource utilization checker captures all the used resources from each cloud sub-module.

## 5. Threat Model

In this paper, we investigated two types of attacks, co-residency and OS attacks, and their impact on VM operation. In a co-residency attack, the attackers exploit their presence on the same host hosting the victim’s virtual machine and start their search for potential vulnerabilities to exploit. For this attack, we used a shuffle-based strategy MtD to minimize the risk values of the VM and the whole host.

OS attacks depend on the vulnerabilities that are exposed within an OS. Attackers track the victim’s VM based on a specific attack path cross-cloud VM allowing attackers to cause damage to the victim VMs. For this attack, we proposed a strategy based on both a shuffle and a diversity MtD minimizing the VM’s risk values.

## 6. The MtD Mathematical Model

The presented system AML relies on the HARM model [23] to analyze the machines’ security metrics. The HARM model divides the networks’ connectivities and vulnerabilities into two layers. The upper layer captures the reachability of the network’s components and the lower layer captures the vulnerabilities existing on each component. The upper layer captures the reachability of the network’s components and is represented by an attack graph (AG) model [27]. The lower layer captures the vulnerabilities existing on each component and is represented by an attack tree (AT).

The attack graph (AG) represents a clear picture of the system risk. It determines if an attacker can reach the final goal state by penetrating the system’s security holes, assuming an initial starting point. For example, if a network consists of two hosts and both have access to the internet, one of the hosts wants to gain access to the other. So, the attacker will try to create a trusted relationship with the target host to gain root access.

The attack tree (AT) is an approach to model an information systems’ security vulnerabilities. It analyzes different security threats, identifies different paths to achieve the goal, and builds a tree structure that describes how a threat helps malicious users to reach their goals. For example, if HostB tries to gain root access to HostA, this would be represented by a multi-stage attack tree. The root of the attack tree is the goal of the attacker. To get root access to HostA, the attacker first needs remote access to the system; therefore, this will be at the next level of the attack tree. The final level of the attack tree will consist of the actions, such as a remote login operation [28].

The AML utilizes HARM to capture the connectivities of VMs on the cloud and OS vulnerabilities existing on each VM to perform the security analysis and compute the security metrics. Such security metrics are the cloud risk (Risk), attack cost (AC), base score (CVSS BS) and return-on-attack (RoA) [22,29].

1 **Cloud Risk**: The risk of each VM is calculated by knowing the values of exploitability $E_{vmi}$ and the impact $I_{vmi}$ of the vulnerability existing on the VM, as shown in Equation (1).
  (1) $$Risk_{vmi}=E_{vmi}*I_{vmi}$$
  Impact (I) shows the exact result that an attacker can cause due to exploiting its vulnerability. Impact metrics are composed of three sub-metrics: confidentiality, integrity and availability.
  Exploitability (E) depends on the characteristics of the vulnerability. Exploitability is composed of four main sub-components: attack vector, attack complexity, privileges required and user interaction.
  In the case of OS attacks, the total risk value on a single VM $Risk_{Total}$ is noted as the sum of all the risk values of other VMs on the attack path passing through the attacker and the targeted VM. In the case of co-residency attacks, the $Risk_{Total}$ will be calculated as the sum of the risk values of all VMs on the same host with the target VM.
2 **Base Score (CVSS BS)**: The base score represents the characteristics of a vulnerability that rely on its impact and exploitability, as shown in Equation (2). The CVSS BS equation for a single VM is represented below
  (2) BaseScore=min(I+E,10)
3 **Attack Cost (AC)**: The AC shows the levels of difficulty of attackers in attacking a system. It represents the cost of exploiting a VM in the cloud for an attacker by considering the attacker’s knowledge of vulnerabilities in the VM. It is calculated as below [30]:
  (3) $$AC_{vmi}=10-BaseScore_{vmi}$$
  In the case of OS attacks, the total attack cost on each VM $AC_{Total}$ is calculated by summing all the AC values of other VMs on the attack path passing through the attacker and the targeted VM. In the case of co-residency attacks, the $AC_{Total}$ will be calculated as the sum of the AC values of all VMs on the same host with the target VM.
4 **Return-on-attack (ROA)**: ROA shows the readiness of the attacker to use the same components, attack paths and vulnerabilities to penetrate the network. The ROA is defined as the ratio of the cloud risk and the attack cost, as shown in Equation (4).
  (4) $$RoA_{vmi}=E_{vmi}*I_{vmi}/AC_{vmi}$$
  Then, the value of the total RoA on a single VM $RoA_{Total}$ is the sum of all the VM ROAs deployed on the same host or passing through the same attack path.

## 7. Discussion and Computational Results

**Figure 4:**
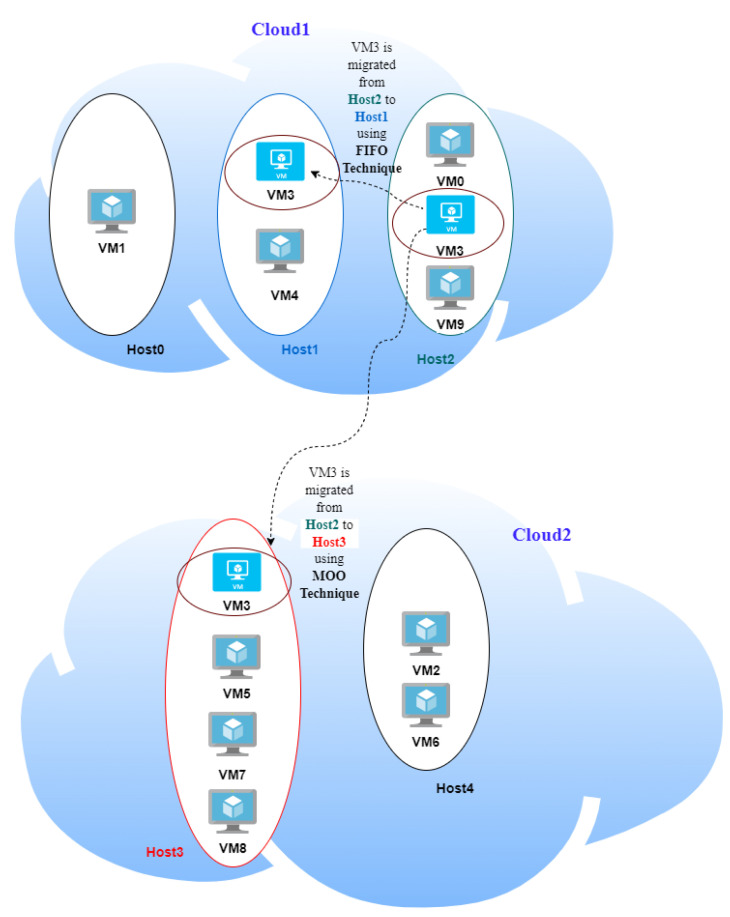
Federated cloud model example.

The computational results show the impact of applying an adaptive moving-target defense model (AML) relying on a self- and situation-aware decision-making mechanism to enhance security measurement levels. A Python-based simulation is used to evaluate the effectiveness of the presented approach to enable security-aware and virtual machine consolidation for efficient resource utilization in the federation. The cloud example model in the evaluation results section assumes two clouds working as a federation; the first has three hosts (Host0, Host1, Host2), while the second has two hosts (Host3, Host4). On these hosts, ten different VMs (VM0, VM1,…, VM9) are distributed, as shown in Figure 4. A primary Linux OS is installed in each VM in the used cloud. Additionally, each VM includes a backup Windows OS. We studied two types of vulnerabilities that threaten the cloud model: side-channel vulnerability and OS-specific vulnerabilities.

To investigate side-channel attacks, we assumed that the CVE-2021-0425 vulnerability [31] was found on the cloud model. Further, for studying OS vulnerabilities, the CVE-2018-14678 Linux OS vulnerability and CVE-2016-3209 Windows OS vulnerability were assumed to exist in the cloud model.

Each vulnerability is represented by its impact (I) and exploitability (E) values that depend on the VM environment. These values are constant across the same user environment, but the values vary among the different VMs as each machine is exposed to different external factors from its operational environment. The values of I and E for each VM are shown in Table 4.

First, we calculated the security metrics; the risk, attack cost, CVSS base score and ROA values for each VM and host, as shown in Table 5.

As discussed, the migration decision is based on the CVSS base score threshold value described in Section 3. The AML finds that Host2 has the highest CVSS base score that exceeds the threshold value, as shown in Table 6. After identifying the host with the highest base score (Host2), all the security metrics values for all its hosting VMs are calculated.

The VM with the highest base score value will be selected to be migrated after checking its criticality level. Table 7 shows the criticality value of each virtual machine

After running all security calculations, VM3 has the highest base score value and the least criticality value. The AML will select the host with minor security metrics to host the migrated VM (VM3). Host1 is found to have the least risk calculation values.

**Figure 5:**
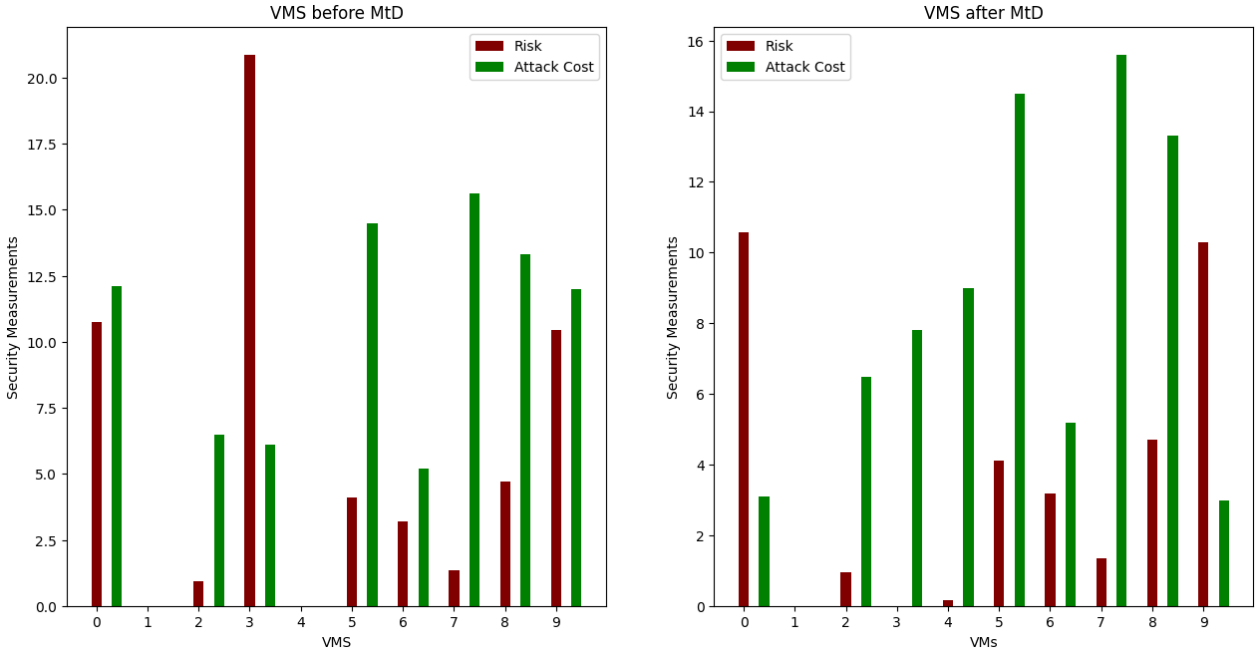
Comparison of security metrics on VMs before and after applying shuffle MtD.

**Figure 6:**
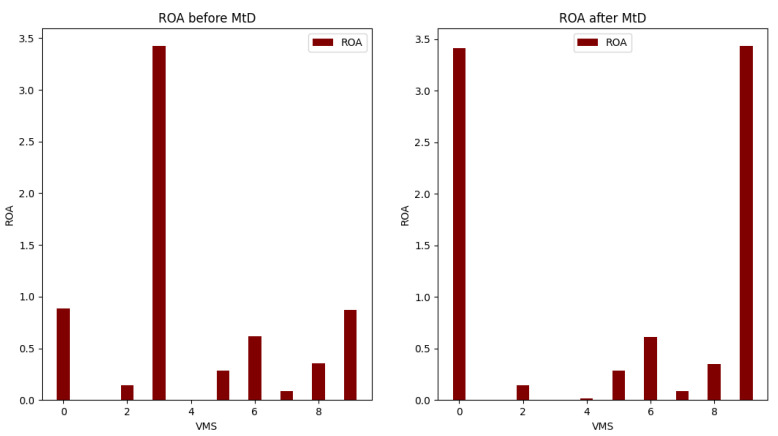
Comparison of ROA before and after applying shuffle MtD.

Table 5 and Table 8 show the calculation results for total risk, total attack cost and return-on-attack (ROA) for each VM before and after applying the shuffle MtD. These values indicate that the risk and ROA values are improved after applying the shuffle MtD techniques. As shown in Figure 5, the total risk of VM3 decreased because the new host (Host1) has a low-risk value. Consequently, the ROA of VM3 is also decreased, which indicates that the probability of attacking this VM is low, as shown in Figure 6.

The risk values for VM0 and VM9 hosted on Host2 are also affected and decreased because of the VM3 migration from Host2, as illustrated in Figure 5 and Figure 6.

If the AML system does not find a host with a low risk level to host the migrated VM, it will select the host with a high resource utilization level. The selection of this host depends on the first in-first out approach (FIFO), meaning that the first host found with a low security risk will be selected.

### 7.1. AML MtD Strategy Design Considering a Multi-Objective Optimization Problem

There is a trade-off between the host security measurement and the resource utilization values. Although Host1 has a lower CVSS base score value, it has a low utilization value.

Consequently, the AML system selects the host that will host the migrated VMs based on a multi-objective optimization problem. The multi-objective optimization problem (MOO) refers to finding a set of optimum solutions, known as the Pareto-optimal front, depending on two or more different objectives.

We started by collecting all non-dominated solution sets, which contain all solutions that are not dominated by any other solution. The non-dominated set of solutions is called the Pareto-optimal front.

After applying MOO, the selected host will be Host3 instead of Host1, which achieves both objectives by achieving high security measurements and better resource utilization value.

We then carried out the same calculation steps to study the impact of OS vulnerability. We assumed that each application is distributed on two machines and that the attack can only come from outside these machines. The application distribution is as follows: VM0 with VM1, VM2 with VM9, VM3 with VM6, VM4 with VM7, and VM5 with VM8.

**Figure 7:**
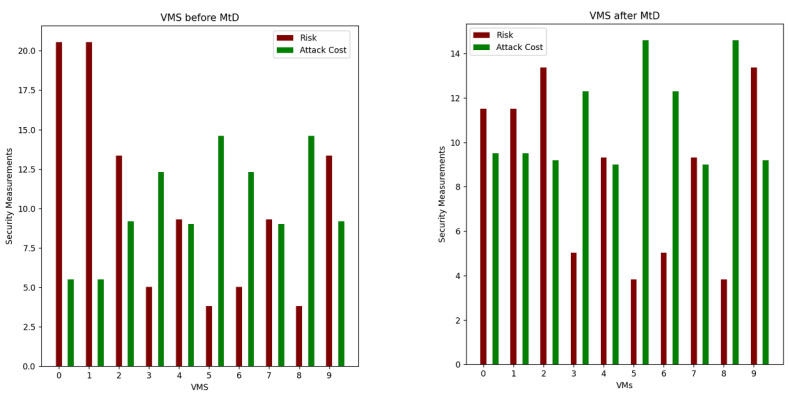
Comparison of security metrics of VMs before and after applying shuffle and diversity MtD.

**Figure 8:**
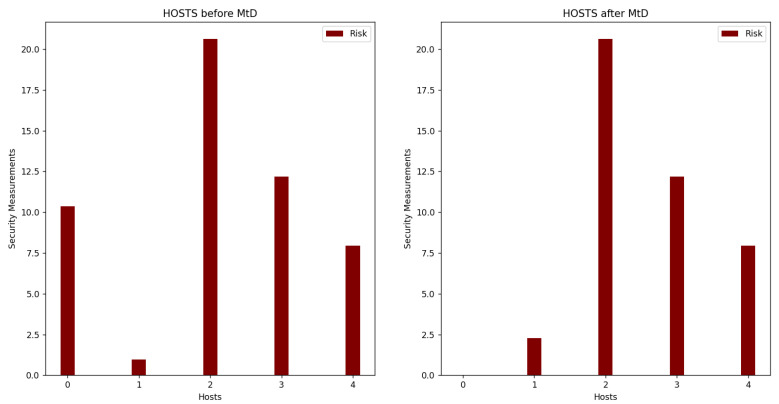
Comparison of security metrics of hosts before and after applying shuffle and diversity MtD.

**Figure 9:**
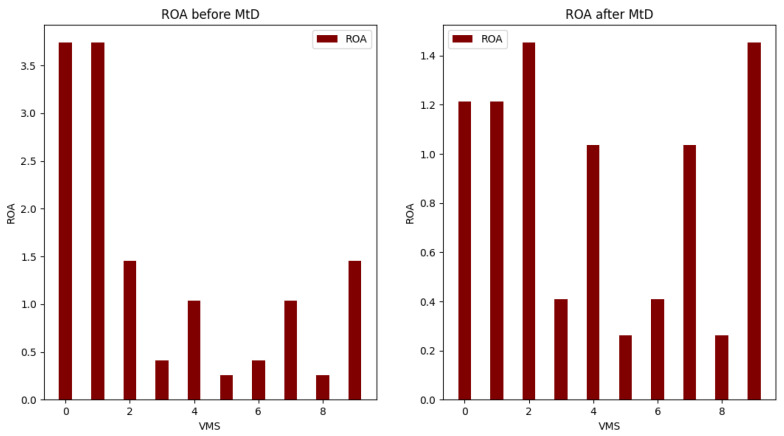
Comparison of ROA of VMs before and after applying shuffle and diversity MtD.

The system starts by calculating all security metrics for all hosts and VMs. For the host exceeding the security metrics threshold, its VMs will be selected for migration. The system will apply OS diversity on VM with the highest base score value. In addition, it will be migrated to another host with lower risk. In this scenario, the migrated VM is VM1 with the highest CVSS base score and will be migrated to Host1. As shown in Figure 7, the risk of VM1 decreased after changing the OS of VM1 and migrating VM1 to Host1. Figure 8 shows the values of the total risk of hosts before and after applying MtD. The total risk of Host0 is now zero due to migrating all its hosted VMs, while the total risk on Host1 has slightly increased due to adding a new hosted VM (VM1). Figure 9 shows the ROA results before and after applying MtD. The RoA of VM1 is decreased due to decreasing its total risk value after applying MtD.

### 7.2. Experimental Design

For the VM migration, we carried out the actual migration of VMs from the OpenStack cloud to OpenNubella. The migration started by taking a snapshot of the migrated machine as an image and then sharing this snapshot with OpenNubella. After that, OpenNubella begins to create a new VM with this image. We carried the migration on a small VM of size 1 GB and it took around three minutes to be operated successfully on the target host.

In order not to cause too many downtimes when repeating the migration process randomly, the AML system takes VM migration decisions depending on any external factors from the VM operational environment, leading to different impact or exploitability values. So, the AML system will repeat the security calculations and trigger the migration decision if any VM has exceeded the threshold values.

## 8. Conclusions and Future Work

This paper presented an adaptive MtD model (AML), a novel virtual machine mobility framework to enable live cross-cloud migrations of running machines for moving-target defense (MtD). The AML relies on the HARM model for cloud security analysis and evaluation. The AML can potentially enhance the cloud’s resource utilization. The AML enables host load-balancing from the perspective of cloud consolidation, which prevents the hosts from operating with limited resource utilization without increasing the risk level on the VMs. The results indicate that the security evaluation of the system was enhanced after applying different MtD techniques, depending on the exposed vulnerability, while efficiently utilizing the host resources. In future work, we intend to generalize the security model to address cloud attacks to enable trustworthy cloud operations in the presence of advanced persistent threats.

## Figures and Tables

**Table 1:** CVSS V3.0 ratings.

Severity	Base Score Range
None	0.0
Low	0.1–3.9
Medium	4.0–6.9
High	7.0–8.9
Critical	9.0–10.0

**Table 2:** Sensitivity levels.

Sensitivity Level	Meaning
0	Low sensitivity
1	High sensitivity

**Table 3:** Criticality levels.

Criticality Level	Meaning
1	Application is not critical
2	The application is less critical as it has two replications
3	The application is critical as it has one replication only
4	Application is very critical and no capsule replication exists

**Table 4:** VMs exploitability (E) and impact (I) in side-channel vulnerability.

VMs ID	I	E
VM0	4.9	2.1
VM1	1.1	1.3
VM2	4	0.8
VM3	0.2	0.8
VM4	2	0
VM5	2.4	0.4
VM6	3.2	0.3
VM7	1.7	2.2
VM8	0.3	1.3
VM9	4.6	2.3

**Table 5:** VMs’ risk, attack cost and ROA before applying shuffle MtD.

VM	Total Risk	Total Attack Cost	ROA
VM0	10.74	12.1	0.88
VM1	0	0	0
VM2	0.96	6.49	0.14
VM3	20.87	6.10	3.42
VM4	0	0	0
VM5	4.13	14.5	0.28
VM6	3.2	5.19	0.615
VM7	1.34	15.6	0.08
VM8	4.7	13.3	0.35
VM9	10.45	12	0.87

**Table 6:** Hosts CVSS base score and utilization values.

Hosts ID	Base Score	Utilization Ratio
Host0	2.4	20
Host1	2.0	20
Host2	4.96	60
Host3	2.76	60
Host4	4.15	40

**Table 7:** VMs’ criticality.

VMs ID	Criticality Value
VM0	3
VM1	2
VM2	3
VM3	2
VM4	1
VM5	3
VM6	2
VM7	4
VM8	1
VM9	3

**Table 8:** VMs’ risk, attack cost and ROA after applying shuffle MtD.

VM	Total Risk	Total Attack Cost	ROA
VM0	10.57	3.10	3.41
VM1	0	0	0
VM2	0.96	6.49	0.14
VM3	0	7.80	0
VM4	0.16	9	0.01
VM5	4.13	14.5	0.28
VM6	3.2	5.19	0.615
VM7	1.34	15.6	0.08
VM8	4.7	13.3	0.35
VM9	10.29	3.0	3.42
